# Dexmedetomidine-Loaded Hydrogel Microneedles Alleviate Acute Inflammatory Visceral Pain in Mice

**DOI:** 10.3390/gels11110904

**Published:** 2025-11-11

**Authors:** Peng Ke, Xin Tan, Yi Zhou, Xiaoyan Bao, Linjie Wu, Min Han, Xiaodan Wu

**Affiliations:** 1 Department of Anesthesiology, Fujian Provincial Hospital, Fuzhou University Affiliated Provincial Hospital, Shengli Clinical Medical College of Fujian Medical University, Fuzhou 350001, China; kepeng@fzu.edu.cn; 2 Institute of Pharmaceutics, College of Pharmaceutical Sciences, Zhejiang University, Hangzhou 310058, China; tanxin@zju.edu.cn (X.T.);; 3 Key Laboratory of Drug Monitoring and Control of Zhejiang Province, National Anti-Drug Laboratory Zhejiang Regional Center, Hangzhou 310051, China

**Keywords:** dexmedetomidine, hydrogel microneedle, analgesia, visceral pain, pain management

## Abstract

Acute inflammatory visceral pain (AIVP) is a prevalent yet challenging clinical condition associated with inflammatory diseases, characterized by diffuse pain that often escalates into nausea, vomiting, and systemic autonomic disturbances. The absence of effective and patient-centered therapies remains a significant clinical challenge. While dexmedetomidine (Dex) has demonstrated promising analgesic effects, its conventional intravenous administration involves slow infusion, heightening risks of infection and compromising patient comfort and compliance. Here, we present a breakthrough strategy using a hyaluronic acid (HA) hydrogel and microneedle-based transdermal system for Dex delivery to enhance clinical practicality. We successfully fabricated Dex-loaded HA hydrogel microneedles (MN/Dex), enabling efficient skin penetration and controlled drug release. Comprehensive biosafety evaluations, including skin irritation, cytotoxicity, and hemolysis assays, confirmed the excellent biocompatibility of the HA hydrogel microneedle system (HA-MN). In the acetic-acid-induced AIVP model, MN/Dex not only produced significant and sustained reduction in visceral and somatic hyperalgesia but also maintained normal physiological activity, avoiding sedation burden, preserving feeding behavior, and supporting natural mobility. MN/Dex offers a minimally invasive, easy-to-administer, and well-tolerated alternative to intravenous therapy, with the potential to transform outpatient management and improve quality of life for patients suffering from AIVP. This advanced delivery platform bridges a critical translational gap in pain management, combining efficacy with outstanding clinical adaptability.

## 1. Introduction

Pain is an early warning signal produced in response to tissue damage, which prompts the organism to avoid danger; it is essential to the process of daily life activities [1]. Prolonged unrelieved pain brings both physical and psychological suffering to the patient [2]. AIVP, caused by damage to internal organs, is commonly seen in acute clinical conditions such as peptic ulcers, perforation, cholecystitis and appendicitis [3], presenting with symptoms including nausea and vomiting, which can be life-threatening in severe cases. Approximately 40% of the global population experience visceral pain [4]. The main analgesic drugs for AIVP include nonsteroidal anti-inflammatory drugs (NSAIDs) and opioids. However, NSAIDs are ineffective for analgesia [5] and opioids carry risks of addiction, constipation and urinary retention, leading to adverse symptoms such as respiratory depression or death [6]. Therefore, traditional analgesic drugs remain unsatisfactory for AIVP.

There is an urgent clinical need for safer and more effective medications to relieve AIVP with fewer concomitant adverse effects. There is also an urgent clinical need for the emergence of analgesic modalities that satisfy the requirements of comfort-oriented medicine, such as patient self-administration. Dex is a good sedative and analgesic [7], whose primary mechanism of action is underpinned by its well-established agonism at α2-adrenergic receptors in the central nervous system. It induces a sleep-like state resembling natural sleep [8] and plays a significant role in enhancing perioperative analgesia and patient comfort [9,10]. Dex is effective in the treatment of AIVP because it inhibits the extracellular signal-regulated protein kinase (ERK) pathway, toll-like receptor (TLR) signaling pathway, transient receptor potential vanilloid 1 (TRPV1) channel [11] and nuclear transcription factor-κB (NF-κB) pathway to attenuate AIVP [12]. Although analgesic and sedative effects can be achieved with intravenous [13], epidural [14], or oral Dex [15], they face disadvantages such as the requirement for continuous intravenous infusion devices, technical difficulties [16], increased risk of neurological damage, and low bioavailability [17], which have led to their notably limited use in AIVP.

HA-MNs, as micron-scale transdermal drug delivery systems, are minimally invasive [18], painless [19], highly bioavailable, and safe [20]. Generally, they are fabricated from biodegradable polymer hydrogels, provide minimally invasive access to interstitial areas, and enable transdermal drug delivery through the gradual dissolution of the drug-loaded needle matrix within the skin [21]. HA, a highly biocompatible endogenous polysaccharide widely present in human tissues, has emerged as an ideal material for HA-MN fabrication due to the excellent viscoelasticity, biodegradability, and biocompatibility of HA hydrogels [22]. HA-MNs have been successfully applied clinically in the treatment of diabetes [23,24], the promotion of hair growth [25], and antitumor activities [26]. They are easy to use and do not touch the nerve fibers in the dermis thereby avoiding pain or nerve damage. Compared to continuous intravenous medication, they are particularly beneficial for improving patient comfort and avoiding catheter-related infections. Transdermal absorption of drugs avoids the first pass elimination effect [27]. Currently, HA-MNs applied for analgesia are mainly used for local analgesia such as for lidocaine microneedles [28]. To date, HA-MNs applied to alleviate AIVP have not been reported.

The development of MN/Dex is undoubtedly beneficial in terms of application to AIVP. MN/Dex patches reduce the adverse effects of conventional drug delivery methods with a simple and rapid delivery method, thereby making it more convenient, effective, and safe to administer, as well as bringing new analgesic measures to bear on visceral pain, including AIVP. In this study, we combined the analgesic effect of Dex and the advantages of HA-MN to prepare drug-loaded MN/Dex. We characterized MN/Dex, demonstrating its favorable safety properties both in vitro and in vivo, as well as its ability to effectively alleviate mechanical, thermal, and visceral pains in AIVP mice. MN/Dex exerted minimal interference on the physiological activities of the mice. The clinical potential of MN/Dex is substantial. If successfully translated, this system could find wide application in various clinical scenarios, including postoperative pain management following relevant surgical procedures, sedation and analgesia in intensive care units, the management of delirium and anxiety, the treatment of insomnia, and even facilitating minor invasive procedures in pediatric or outpatient settings where patient cooperation is challenging. By establishing the foundational efficacy and safety evidence for this novel delivery approach, our study serves as a crucial first step in bridging the gap between laboratory research and clinical application, paving the way for realizing these significant clinical benefits.

## 2. Results and Discussion

### 2.1. Fabrication and Characterization of MN/Dex

AIVP is highly prevalent in a variety of clinical disorders, including inflammatory bowel disease [29]. Although significant progress has been made in understanding somatic pain, research on visceral pain including AIVP remains limited [30]. Currently, no specific therapeutics are available for AIVP. In recent years, Dex has been found to have satisfactory analgesic effects on visceral pain [31]. However, the disadvantages of open intravenous access and continuous intravenous pumping restrict its widespread utilization [32].

**Figure 1:**
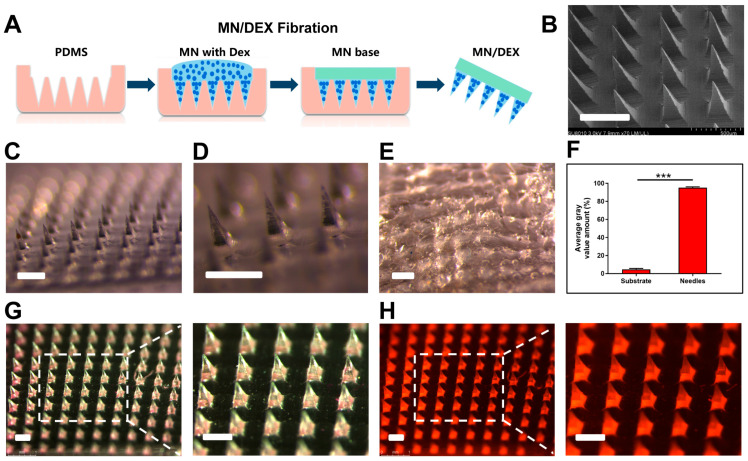
Drug in the microneedle was mainly distributed in the needle body. (**A**) Preparation process of MN/Dex. (**B**) SEM image of MN/Dex; drug distribution. (**C**,**D**) Fabricated MN/Dex and (**E**) dissolved MN/Dex under the stereoscopic microscope. (**F**) The amount of Rhodamine B fluorescence molecules loaded into the MNs. *** indicates statistical significance of gray value between the group “Substrate” and “Needles” at the level of *p* < 0.001 using Student’s *t*-test; n = 3/group. (**G**) The images of Rhodamine B microneedles. (**H**) The confocal fluorescence microscopy images of Rhodamine B microneedles. All scale bars represent 500 µm.

To address this limitation, we developed MN/Dex patches for the effective transdermal delivery of Dex. The MN/Dex patches were fabricated using a casting method, with the backing body stock solution consisting of drug-free polyvinyl pyrrolidone K90 (PVP K90) (Figure 1A). The microneedle mold had a volume of 1.996 µL, and the concentration of Dex in the microneedle body stock hydrogel solution was 2 mg/mL, resulting in a drug loading of approximately 4 μg per patch. The therapeutic efficacy of MN/Dex in treating AIVP depends on proper microneedle quality. Morphological characterization using a stereoscopic microscope and scanning electron microscope (SEM) confirmed a well-defined microneedle structure, consistent with established evaluation methods [33]. Each MN/Dex patch contained a 15 × 15 array of uniformly arranged microneedles with regular morphology. The microneedles measured 220 µm in side length, 550 μm in height, and 500 μm in adjacent microneedle center-to-center spacing, which is consistent with the dimensions of the microneedle mold (Figure 1B,D). The quality assessment confirmed the well-defined structure of the microneedle with concentrated drug distribution in the needle bodies.

After application to mouse skin, MN/Dex completely dissolved without leaving residual needles in the backing substrate (Figure 1E). Fluorescence imaging of Rhodamine B microneedles (model drug microneedles) showed 95.27% drug localization in the needle bodies (Figure 1F), with the remaining 4.73% attributed to backing solution residue from the preparation process. Confocal microscopy revealed neatly arranged Rhodamine B microneedles under a bright field (Figure 1G), while dark field imaging demonstrated intense red fluorescence concentrated in the needle body portion, particularly at the base (Figure 1H), confirming that the drug in MN/Dex was mainly distributed in the needle body.

### 2.2. Mechanical Strength and Transdermal Drug Release Capability of MN/Dex

**Figure 2:**
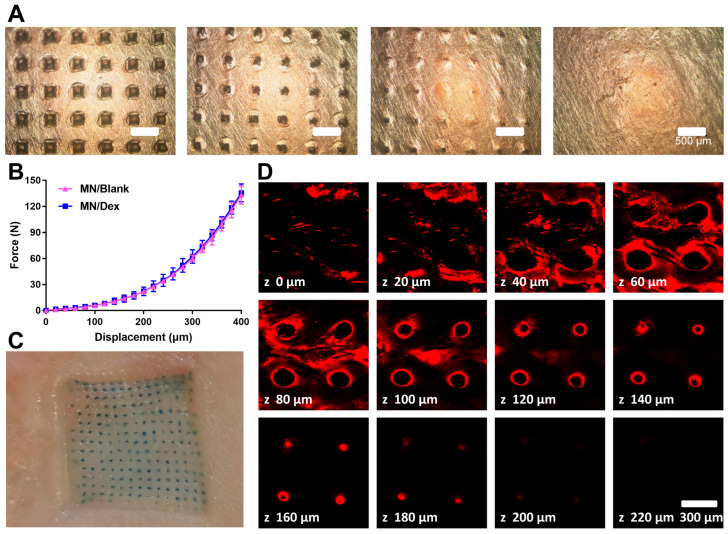
Microneedles possess sufficient mechanical strength to penetrate the skin. (**A**) Penetration experiment of MN/Dex passes through Parafilm^®^ M sealing film. Scale bar = 500 µm. (**B**) Mechanical strength changes in MN/Dex compressed under universal material testing machine. n = 3/group. (**C**) Transdermal situation of the microneedle of trypan blue model, where each blue spot indicates a successful penetration by a single microneedle. Scale bar = 1 mm. (**D**) Depth of drug distribution by microneedle puncture under the confocal laser scanning microscope. Scale bar = 300 µm.

Penetration of the stratum corneum of the skin is a basic requirement for microneedling. HA hydrogel serves as an ideal microneedle material due to its optimal viscoelasticity, which enables rapid and safe in vivo dissolution while maintaining sufficient sharpness and mechanical strength for skin penetration. Previous reports indicate that a force of 580 mN enables effective microneedle penetration [34]. In a penetration test using a 500 μm thick four-layer Parafilm^®^ M sealing film, MN/Dex easily penetrated the first two layers, leaving clearly visible square holes (Figure 2A). The universal material testing machine test showed a total MN/Dex patch mechanical strength of 135.67 N, corresponding to approximately 600 mN per needle (Figure 2B).

In a drug delivery assessment, Taipan blue microneedles (model microneedles) formed a regularly arranged array of blue dots on skin after application, with over 93% of the microneedles leaving visible pores. This result visually confirms the successful skin penetration of MN/Dex indirectly (Figure 2C). Rhodamine B, which has a molecular weight similar to Dex, was used to evaluate drug delivery depth. The red fluorescent signal was dominantly distributed in 20–100 μm, with some signal detectable up to 200 μm (Figure 2D). These findings demonstrate that MN/Dex rapidly dissolves within skin tissue, releasing the drug within two minutes after application. The combination of skin puncture experiments and depth-dependent fluorescence images confirms the favorable transdermal drug delivery capability of the HA-MN.

### 2.3. Biosafety Evaluation of MN/Dex In Vivo and In Vitro

**Figure 3:**
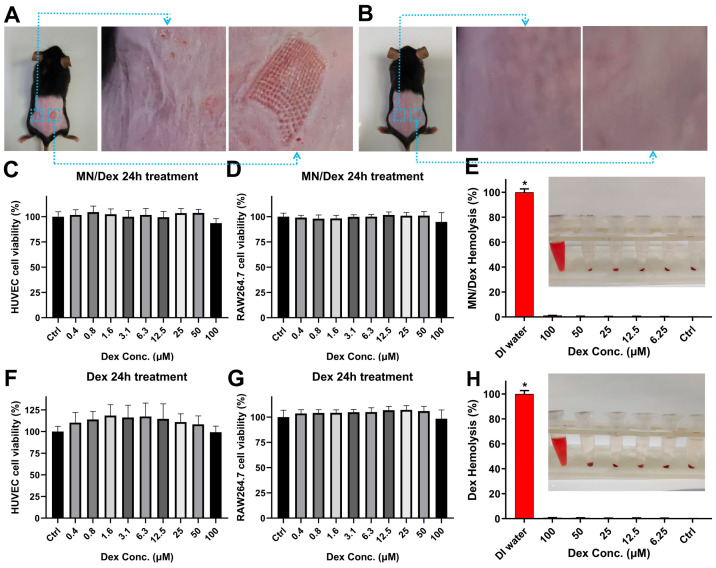
MN/Dex exhibits favorable safety profiles in vivo and in vitro. (**A**) Skin surface of the mouse after removal of the backing of microneedles. (**B**) The surface of mouse skin after 3 h of using microneedles. (**C**) Toxicity of MN/Dex in HUVECs and (**D**) RAW 264.7. n = 6/group. (**E**) Cytolytic situation and hemolysis rate after MN/Dex application. n = 3/group. * indicates a level of *p* < 0.05. (**F**) Toxicity of Dex in HUVECs and (**G**) RAW 264.7. n = 6/group. (**H**) Cytolytic situation and hemolysis rate after Dex application. n = 3/group. Statistical significance for intergroup comparisons was analyzed by one-way ANOVA with a post hoc test. * *p* < 0.05 versus the control group. Data are shown as mean ± SD.

The safety of MN/Dex is the focus of our concern and determines its application in AIVP treatment. The HA hydrogel, a natural skin component, has established skin compatibility and offers favorable hydration, adhesion, and wound healing properties for dermatological applications [35]. In this study, the MN/Dex patch left a clearly visible pinholes on the skin surface and traces of pressed skin on the contralateral side (Figure 3A). The skin recovered completely within three hours (Figure 3B), demonstrating the excellent skin compatibility of HA-MN.

Cytotoxicity assays and hemolysis assays are the classic methods for evaluating drug safety in vitro [36]. Human umbilical vein endothelial cells (HUVECs) are human-derived normal cells [37] and mouse monocyte-macrophage leukemia cells (RAW 264.7) are murine-derived immune cells. MN/Dex stock hydrogel solution showed no cytotoxic effects on either RAW 264.7 cells or HUVECs (Figure 3C,D), confirming the good biosafety of MN/Dex. Hemolysis experiments revealed minimal red blood cell damage, with a hemolysis rate of 1.25% at the highest MN/Dex concentration and below 1% for all other samples (Figure 3E). All values remained well under the 5% of the national standard, supporting the biosafety and circulatory system compatibility of the formulation [38]. Additionally, as an approved pharmaceutical agent, Dex showed no significant cytotoxicity or hemolytic activity in our experimental assessment (Figure 3F–H). Together, these findings support the favorable safety of MN/Dex for translational applications.

### 2.4. Analgesic Effects of MN/Dex in AIVP Mice

**Figure 4:**
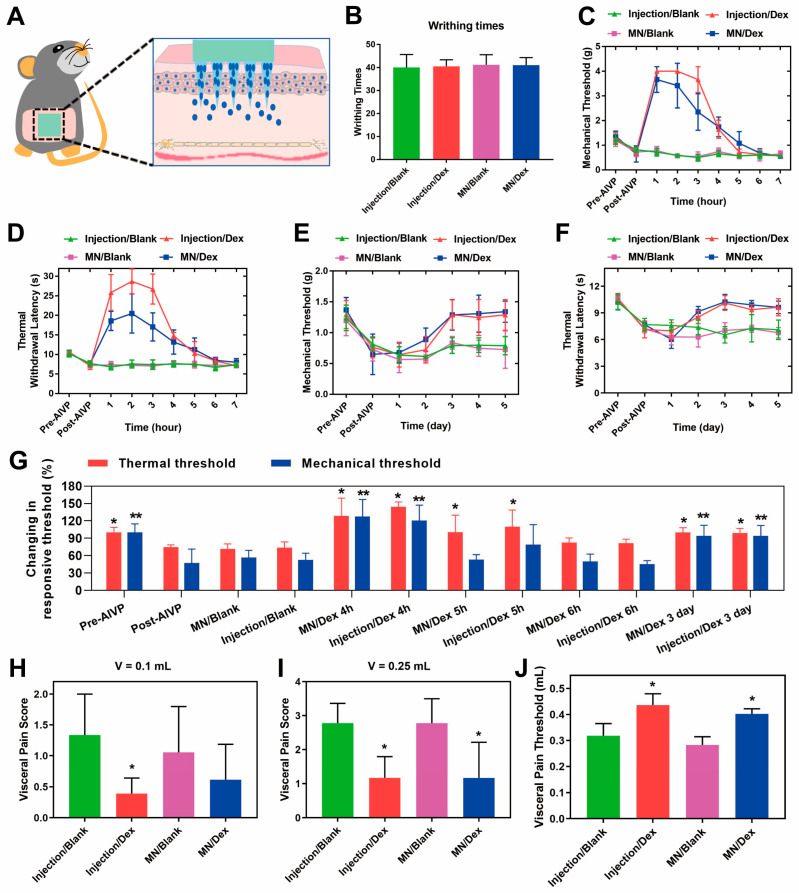
MN/Dex alleviates mechanical, thermal, and visceral pain in AIVP mice. (**A**) Schematic diagram of drug administration. (**B**) Amount of writhing in mice. (**C**) Changes in mechanical pain and (**D**) thermal pain on the day of drug administration. (**E**) Continuous mechanical pain and (**F**) thermal pain changes after drug administration. (**G**) Comparison and summary of pain threshold changes at different time points under experimental conditions. Data are presented as mean ± SD (n = 6). * *p* < 0.05 versus the “Post-AIVP (Thermal threshold)” group; ** *p* < 0.05 versus the “Post-AIVP (Mechanical threshold)” group (one-way ANOVA followed by a post hoc test). (**H**) Visceral pain on day 3 after drug administration with nociceptive score at 0.1 mL and (**I**) 0.25 mL and (**J**) visceral nociceptive threshold. Statistical significance for intergroup comparisons was analyzed via one-way ANOVA with a post hoc test. * *p* < 0.05 versus the Injection/Blank group. Data are shown as the mean ± SD (n = 6).

The transdermal delivery of Dex via MN/Dex into the circulation exerted effective systemic analgesia (Figure 4A). We established the acetic-acid-induced AIVP model, a validated stable model of visceral pain [39], in which mice developed characteristic writhing responses, confirming successful modeling (Figure 4B). The Von Frey and hot-plate experiments are classic, non-invasive methods for testing somatic pain repeatably [40,41]. The reduction in mechanical and thermal pain thresholds observed in AIVP mice signifies nociceptive hypersensitivity, thereby validating the efficacy of the modeling approach.

Both the MN/Dex and subcutaneous Dex group (Injection/Dex) treatments significantly reversed AIVP-induced mechanical pain hypersensitivity, with comparable analgesic efficacy between the two delivery routes (Figure 4C). Notably, the pharmacodynamic equivalence observed between the MN/Dex and Injection/Dex groups, evidenced by nearly identical pain relief profiles, provides compelling functional evidence that renders direct pharmacokinetic measurement less critical for establishing therapeutic efficacy.

Furthermore, we observed a difference in the pharmacodynamic profiles between the two administration groups. Injection/Dex produced analgesia exceeding baseline thresholds, suggesting transient oversedation from rapid drug delivery, while MN/Dex provided more moderated and sustained pain relief without apparent oversedation. This differential effect aligns with the distinct release kinetics of each formulation. The drug released from the HA-MN must first diffuse through the stratum corneum and viable epidermis before reaching the dermal microcirculation for systemic absorption.

Acting as a drug reservoir within the skin, the dissolving HA-MN provides the sustained release of Dex as the polymer hydrates and dissolves, thereby increasing the drug’s local residence time. This process, governed by dissolution and diffusion kinetics, inherently introduces a lag time before therapeutic concentrations are achieved in the central nervous system. Consequently, this results in a flatter and more prolonged plasma concentration–time profile, which directly translates into the sustained analgesic effect we observed, while potentially offering a superior safety profile by avoiding acute peak concentrations associated with oversedation.

Similarly, both MN/Dex and Injection/Dex effectively relieved thermal pain, with a longer-lasting effect compared to mechanical pain relief (Figure 4D). This comparable duration and magnitude of the analgesic effect strongly suggests that the MN/Dex system achieves systemic drug delivery that is sufficient to produce therapeutic concentrations at the target sites. The sustained therapeutic effect was further demonstrated by the gradual normalization of mechanical and thermal pain thresholds of mice in the MN/Dex and Injection/Dex groups over five days after AIVP, with mechanical and thermal hypersensitivity completely resolving by the third day in both treatment groups (Figure 4E,F), suggesting that Dex was beneficial in alleviating the hypersensitivity of mechanical and thermal pain in AIVP mice. Both the MN/Dex and Injection/Dex groups exhibited sustained analgesic effects. Significant thermal analgesia was maintained for over five hours post-administration, and mechanical analgesia persisted for at least four hours, compared to the Post-AIVP group. Furthermore, both thermal and mechanical pain thresholds remained improved in these two groups at the three-day follow-up, indicating a durable therapeutic effect (Figure 4G). Additionally, colorectal distension tests on day 3 revealed significantly improved visceral pain threshold in both MN/Dex and Injection/Dex groups, requiring greater stimulation volumes to elicit pain responses compared to controls (Figure 4H,I).

At the same time, more stimulation was required to reach a nociception score of 3 in the MN/Dex and Injection/Dex groups (Figure 4J), suggesting that Dex ameliorated visceral pain hypersensitivity. No analgesic effect was observed in subcutaneous saline group (Injection/Blank) and blank microneedles (MN/Blank). For transparency and reproducibility, exact *p*-values and confidence intervals are reported for all statistical comparisons in the Supplementary File (Table S1). The observation that MN/Dex and Injection/Dex at the same nominal dose produce essentially superimposable pharmacodynamic responses provides compelling indirect evidence that the transdermal system achieves biologically equivalent drug exposure. This functional equivalence, demonstrated through multiple validated pain models, underscores the translational potential of MN/Dex while rendering direct blood concentration measurements redundant for establishing therapeutic efficacy.

### 2.5. Effects of MN/Dex on Physiological Behavior in Mice

**Figure 5:**
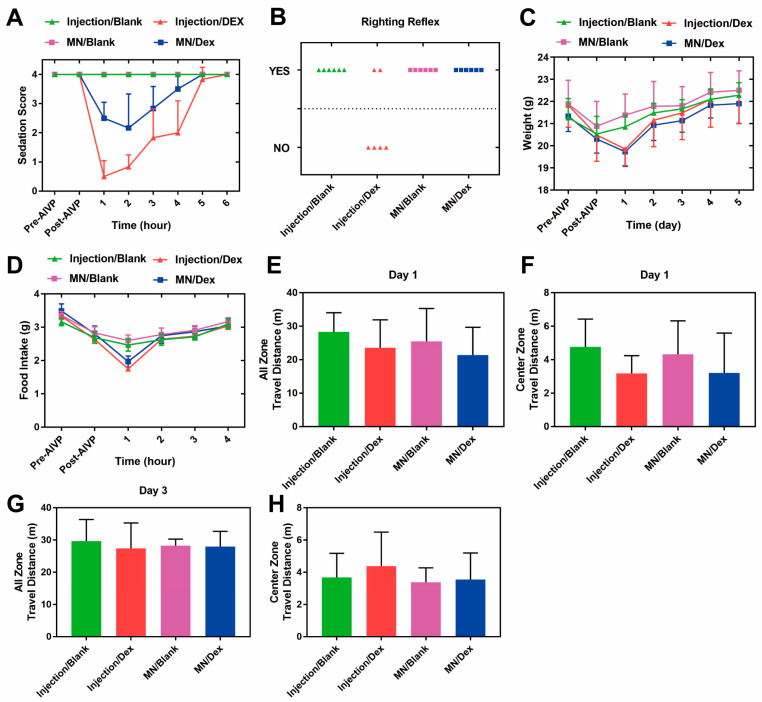
MN/Blank causes no impairment of physiological behavior in mice. (**A**) Sedation scores after drug administration. (**B**) Righting reflex. (**C**) The changes in body weight and (**D**) food intake in mice. (**E**) Total motion distance and (**F**) central zone motion distance of mice on the first day in Open-field experiment. (**G**) Total motion distance and (**H**) central zone motion distance of mice on the third day in Open-field experiment. n = 6/group. Statistical significance for intergroup comparisons was analyzed by one-way ANOVA with a post hoc test. Data are shown as the mean ± SD.

Dex induces sedation similar to natural sleep, which is beneficial for helping pain patients to endure torturous moments [42]. Based on this, we evaluated the effects of MN/Dex on sedation scores and even anesthesia in mice [43,44]. Lower sedation scores indicate a deeper level of sedation in the animal. Compared with the Injection/Dex group, the MN/Dex group had a higher sedation score 5 h after administration, indicating that microneedle administration was beneficial in alleviating the nonessential sedative effects of Dex (Figure 5A). Four mice in the Injection/Dex group lost their righting reflex and had deeper sedation (Figure 5B), suggesting that MN/Dex administration prevents the occurrence of nonessential over-sedation. Dex is prone to inducing nonessential deep sedation [45]. The mice in the MN/Dex group all remained awake, which may be related to the slower absorption at the microneedle release site or the slow release of Dex facilitated by the viscous wrapping of sodium hyaluronate [46,47]. Body weight and food intake reflect the physical condition of the mice [48], and the distance traveled in the open field reflects the anxiety of the mice [49]. The observed decrease in body weight after modeling was attributed to AIVP-induced reduction in food intake. In Dex-treated mice, the weight loss was associated with the sedative effect of the drug, which also led to decreased food consumption. However, no statistically significant differences in body weight or food intake were observed among the groups after two days of administration (Figure 5C,D), indicating that Dex did not exert prolonged effects on body weight or feeding behavior in mice. The open-field experiments showed no statistically significant differences in total locomotor distance and central zone locomotor distance between groups of mice (Figure 5E–H), demonstrating the safety of MN/Dex without impairing long-term locomotor performance or anxiety levels in mice.

This study represents a breakthrough in the routine administration of Dex and an exploration of future clinical applications of Dex microneedles. We respectfully acknowledge several aspects that contextualize our work. Firstly, while the analgesic effects of Dex have been reported [50], the principal innovation of this study lies in the development of a microneedle-based delivery platform, an advancement that addresses key practical limitations of conventional administration routes. Our study primarily focuses on innovating its delivery route rather than discovering a new pharmacological action. Secondly, regarding the absence of pharmacokinetic analysis, this reflects a deliberate and scientifically motivated study design. Given the exceptionally low drug loading of 4 µg in MN/Dex and the large volume of distribution in mice (>2 L/kg), the theoretical peak plasma concentration would be prospectively estimated to be approximately 80 pg/mL. This concentration is near or below the quantitation limit of even advanced analytical methods such as liquid chromatography–tandem mass spectrometry (LC-MS/MS). In light of this technical constraint, we strategically focused on establishing clear pharmacodynamic proof-of-concept, which we successfully achieved through robust in vivo efficacy data. Finally, the MN/Dex delivery system was successfully constructed and validated for the treatment of AIVP, demonstrating both therapeutic feasibility and a favorable safety profile. This achievement not only provides a promising strategy for managing AIVP but also establishes a critical foundation for expanding the clinical applications of Dex through transdermal delivery. The research serves as an essential bridge from laboratory research toward future clinical translation, paving the way for broader implementation of this innovative drug delivery platform.

For future clinical translation, several key aspects of the MN/Dex patch have been addressed. The solvent-casting fabrication method supports scalable production using industrial processes such as continuous molding and automated filling, with commercially available pharmaceutical-grade HA and PVP facilitating manufacturing. Sterilization via gamma irradiation or ethylene oxide is feasible for the dried patches, as these methods are compatible with hydrogel systems and preserve mechanical and drug properties. Furthermore, the patch stored in sealed packaging at room temperature can be stored for at least 12 months. These features underscore the translational potential of MN/Dex for practical clinical use.

## 3. Conclusions

Our study utilized a novel delivery modality for delivering Dex. Our findings demonstrate that MN/Dex patches effectively exert the effect of alleviating pain symptoms in AIVP mice with minimal side effects, providing a research basis and insight for the application of microneedle transdermal drug delivery strategies to other types of visceral pain and clinical translation.

## 4. Materials and Methods

### 4.1. Reagents and Materials

The list of regents was obtained commercially. The polydimethylsiloxane microneedle mold was acquired from Guangzhou Shiling Laike Mold Trading Co., Ltd. (Guangzhou, China). HA, (10–100 kDa) was acquired from Shandong Huaxi Biotechnology Co., Ltd. (Jinan, China). Dex was acquired from Shanghai Yuanye Biotechnology Co., Ltd. (Shanghai, China). PVP K90, Rhodamine B and the Tai-Pan Blue Staining Kit were acquired from Shanghai Aladdin Reagent Co., Ltd. (Shanghai, China). Parafilm^®^ M sealing film was acquired from Merck KGaA (Darmstadt, Germany). The CCK-8 reagent kit was purchased from Shanghai Beyutime Biotechnology Co., Ltd. (Shanghai, China).

### 4.2. Use of Microneedle Molds

Each piece of microneedle mold contained 225 holes of uniform size (microneedle arrays were arranged in 15 × 15 rows), with a depth of 550 µm, a side length of 220 μm, and a center distance of 500 μm between adjacent holes. The microneedle molds were cleaned sufficiently before use each time by immersing the molds in a beaker of ultrapure water for 30 min, followed by placing the beaker into an ultrasonic cleaner (parameter set at 80 Hz) for 10 min. The molds were dried in a thermostatic blower at 60 °C for 1 h after drying [51].

### 4.3. Preparation of Hydrogel Microneedles

For the preparation of MN/Dex, Dex powder was dissolved in deionized water to form a Dex solution at a concentration of 2 mg/mL, and then, HA powder was added to formulate a 250 mg/mL MN/Dex body stock hydrogel solution, which was allowed to stand at room temperature and dissolved overnight until the hydrogel solution was transparent, homogeneous, and free of air bubbles. Another PVP K90 was dissolved in deionized water to a concentration of 250 mg/mL as the MN/Dex backing substrate, which was left at room temperature and dissolved overnight until the backing substrate was transparent, homogeneous, and bubble-free. The MN/Dex was fabricated using the casting method [52]. Firstly, the body stock solution (approximately 0.1 mL) was injected into the microneedle mold, which was centrifuged at 3500 rpm for 5 min, and the body stock solution was scraped off the surface of the microneedle mold and dried in a desiccator for 15 min. Then, the backing substrate liquid was added until the liquid surface reached the edges of the microneedle mold, before being centrifuged at 3000 rpm again for 1 min. The centrifuged molds were placed in a desiccator and left to dry and demolded at room temperature for 24 h to obtain regular, intact MN/Dex.

For the preparation of MN/Blank, Dex was not present in the microneedles. Taipan blue microneedles and Rhodamine B microneedles were used as model drug microneedles to mimic the properties of MN/Dex [53], which were prepared using the same method as that of MN/Dex. The Dex solution was replaced by 0.4% Tepan blue solution for Tepan blue microneedles and 1 mg/mL Rhodamine B solution for Rhodamine B microneedles, and the rest of the procedure was the same as that for MN/Dex.

### 4.4. Characterization of the Morphology and Structure of Microneedles

For observation under a stereoscopic microscope, we gently held both sides of the microneedle with sharp forceps to fix the microneedle without deforming it, before placing the microneedle on the stage of the stereoscopic microscope (Nikon Corporation, Tokyo, Japan) and adjusting the coarse and fine focusing helix, as well as the angle of the light, to observe the microneedle morphology from the appropriate position and angle. Photographs were obtained under optimal imaging conditions.

For observation under a scanning SEM (HITACHI, Tokyo, Japan), the microneedles were carefully fixed in the sample stage with conductive adhesive, placed in a vacuum coater, and imaged using a SEM after sputtering gold and palladium on the microneedle surface. The lens was adjusted to focus on the sample, and then the field of view and magnification were adjusted to the appropriate imaging conditions, and then the SEM image of the microneedle surface was taken. Microneedle imaging was recorded using a FEI/Philips XL30 FEG ESEM system (FEI Company, Hillsboro, OR, USA).

A confocal laser scanning microscope (Leica, Mannheim, Germany) was employed to indirectly visualize the distribution of the drug within the body and base of the microneedles. The equipment was operated according to the specifications, the 540 nm excitation spectrum was set, and the 580 nm emission spectrum was collected. Subsequently, the Rhodamine B microneedle was gently fixed on the microscope stage and adjusted to the center of the field of view and focused. Images under fluorescence and bright field were then recorded.

### 4.5. Characterization of the Mechanical Strength of Microneedles

A Parafilm^®^ M sealing film puncture test and universal material testing machine test were conducted to assess the mechanical strength of microneedles. For the Parafilm^®^ M sealing film puncture test, multiple layers of Parafilm^®^ M sealing film were stacked to simulate skin [54] and investigate MN/Dex permeation. After neatly stacking 5 layers of Parafilm^®^ M sealing film, MN/Dex was used to puncture and observe the holes left on the surface of the film.

For the universal material testing machine test, the microneedle is secured and placed in the center of the universal material testing machine horizontal stage, with the tip of the needle facing the sensing probe and retained a small distance. The probe is moved downward at 0.01 mm/s while the sensor records mechanical data 200 times per second. Pressure and displacement were zeroed when the probe came into contact with the tip of the needle. The probe was then moved to obtain pressure–displacement data and plotted to obtain a pressure versus displacement curve.

### 4.6. Skin Puncture Experiment

The muscle and adipose tissue on the back skin of euthanized mice were removed and drained with absorbent paper. The skin of the mice was placed face up. A microneedle of Taipan blue was inserted into the skin of the mice and removed after 2 min of finger pressure. The penetration of the microneedle patch into the skin was determined by observing the holes formed in the skin by drying the skin surface with a cotton ball.

### 4.7. Confocal Laser Scanning Microscope Scanning Skin

Drug distribution after microneedle transdermal use was examined using a confocal laser scanning microscope [55]. Rhodamine B microneedles served as a model drug to reflect the distribution of MN/Dex in the skin after application. The microneedles were applied to the skin of mice for 2 min, then removed, and immediately imaged with a confocal microscope. The excitation wavelength was set at 550 nm, the emission wavelength at 580 nm, and the *z*-axis scanning layer was scanned to determine the distribution of the drug.

### 4.8. Skin Irritation Test

MN/Dex was applied to the dorsal skin of mice for 2 min and then removed. The changes in the skin on the microneedle side and the contralateral side were observed 3 h later.

### 4.9. RAW 264.7 and HUVECs Culture

Mouse monocyte macrophage line RAW 264.7: RAW 264.7 cells (National Collection of Authenticated Cell Cultures, Shanghai, China) were cultured in culture flasks placed in a constant temperature cell culture incubator at 37 °C with 95% O_2_ and 5% CO_2_. RAW 264.7 passages were decellularized using a disposable sterile cell scraper instead of trypsin. Cells were cultured in Roswell Park Memorial Institute (RPMI-1640, Thermo Fisher Scientific, Waltham, MA, USA) medium containing 10% fetal bovine serum. The medium was refreshed every two days.

Culture of HUVECs: the method for HUVEC culturing differed from RAW 264.7 in that 0.25% trypsin was used for digestion of the cells during passaging. The rest of the procedure was the same as for the culture of RAW 264.7.

### 4.10. Cell Counting Kit-8 Assay

After taking RAW 264.7 cells and resuspending them, the cells were observed under the microscope and counted to prepare a cell suspension with a density of 1 × 10^5^ cells/mL. The cell suspension was transferred to a 96-well plate, with each cell receiving 100 µL of cell suspension. After 6 h, when the cells grew adherently to the wall, different concentrations of the drug were added to each well. The concentrations of Dex were 0, 0.39, 0.78, 1.56, 3.13, 6.25, 12.5, 25, 50, and 100 µM, and the corresponding HA concentrations were 0, 9.2, 18.4, 36.9, 73.9, 147.9, 295.9, 591.8, 1183.7, and 2367.4 μg/mL, and six replicate wells were set up in each concentration group. The experimental procedures of HUVECs were the same as those described above, except that the concentration of cell suspension was 3 × 10^4^ cells/mL at the time of seeding the plate. In addition, we also set up an experiment with only Dex added as a negative control. Cell activity was assessed using the CCK-8 assay, where 24 h after administration, the supernatant was discarded, and each well received 100 µL of culture medium containing 10 μL of CCK-8 solution. The cells were then further incubated for 1–3 h, and absorbance at 450 nm was measured at the appropriate time using an enzyme marker. Cell survival rate = (experimental wells − blank wells)/(control wells − blank wells) × 100%.

### 4.11. Hemolysis Assay

In brief, 2 mL of fresh mouse blood was collected into EP tubes (sodium heparin moistened tubes) and centrifuged at 3000 rpm for 5 min at 4 °C. The supernatant was discarded and washed three times with PBS until it was clear and transparent and then resuspended to a 2% erythrocyte suspension by adding PBS. The preparations were grouped into Dex and MN/Dex groups. The concentration of Dex in the two groups was set at 6.25, 12.5, 25, 50 and 100 µM. We also set up the group without Dex as a negative control. Each sample received 0.5 mL of suspension mixed with 0.5 mL of the respective preparation and was then incubated at 37 °C for 4 h. After incubation, the tubes were centrifuged at 4 °C for 1 min at 15,000 rpm, and the changes in the supernatant were observed and photographed. The supernatant of each tube was transferred to a 96-well plate, and the absorbance of the supernatant at 540 nm was measured with an enzyme meter. Deionized water was set as positive control with a hemolysis rate set as 100%, and PBS erythrocyte suspension was set as negative control. The hemolysis rate was calculated from the absorbance value of each sample. The formula was calculated as follows: hemolysis rate = (sample − negative control)/(positive control − negative control).

### 4.12. Animals

Five-week-old male C57BL/6 mice obtained from the Laboratory Animal Center of Fujian Medical University were used for the animal experiments. All animal experiments were approved by the Experimental Animal Ethics Committee of Fujian Medical University (IACUC FJMU 2022-0891, Fuzhou, China) and in accordance with the World Medical Association Declaration of Helsinki. All mice were housed in pathogen-free cages, allowed to move freely, and provided with autoclaved food and water. In addition, mice were kept at controlled temperature (22 ± 1) °C and humidity (55 ± 10%). They were shaved and depilated under sevoflurane anesthesia after 2 weeks of acclimatization feeding. On the second day, 24 mice were randomly assigned to 4 groups using a computer-generated random number sequence before the successful establishment of the AIVP model: Injection/Blank, Injection/Dex, MN/Blank, and MN/Dex group. Six mice were allocated to each group. AIVP model was established by intraperitoneal injection of 0.7% acetic acid solution (at a dose of 10 mL/kg) into mice. Mice with a concave abdomen, anterior abdominal wall close to the bottom of the cage, twisted buttocks and backward extension of hind limbs showed the special posture of twisted body reaction, indicating successful model establishment. The mice were then immediately placed into the observation box for observation, and the number of times the abdomen was concave, and the hind limbs were stretched was counted as one torsion; the number of torsions that occurred within 30 min was counted as an index for evaluating the establishment of the AIVP model. All behavioral assessments (including pain tests) were conducted by an experimenter who was blinded to the group allocation of the animals throughout the entire data collection and analysis phase. The treatments were administered by a separate researcher. The drugs were administered immediately after assessing peripheral pain, and the same administration schedule was followed the next day, resulting in a total of two administrations. In the experiment, 50 µL of saline was injected subcutaneously in the Injection/Blank group; 50 μL of Dex (180 μg/kg) was injected subcutaneously in the Injection/Dex group. The MN/Dex group was treated with the use of one MN/Dex patch (4 μg/patch, which is 180 μg/kg). The coded group assignments were only revealed after all behavioral data had been recorded.

### 4.13. Mechanical Nociception Test

Mechanical pain in the mice was assessed using Von Frey fibers [39]. Mice were gradually acclimatized and kept quiet inside a transparent glass with a wire mesh bottom. We applied Von Frey fibers (0.04, 0.07, 0.16, 0.4, 0.6, 1.0, 1.4, 2.0, and 4.0 g) vertically to the middle part of the plantar side of the mice, starting with a force of 0.4 g and following the up-and-down method. The fiber filaments contacted the plantar foot in a slightly curved shape for 2–3 s until the animal lifted its foot or fled away. A negative response of foot contraction or foot licking was recorded as “O”, and a positive response of foot contraction or foot licking was recorded as “X”. The 50% foot-contraction response threshold was calculated by obtaining a sequence of “O” or “X” combinations from five consecutive stimuli.

### 4.14. Thermal Pain Test

The temperature of the hot plate was set to 55 ± 0.5 °C. After heating to the set temperature, the mice were placed on the hot plate for the experiment. The time for the mice to appear to lick hind feet on the hot plate was recorded as the thermal pain latency. Each mouse was evaluated three times, each time 1 min apart, and the mean value was calculated.

### 4.15. Abdominal Withdrawal Test

Visceral pain in mice was assessed by observing the threshold intensity of the abdominal withdrawal reflex as described by Lu Ying et al. [36]. After 2% sevoflurane anesthesia in mice, a 4F double-lumen catheter was inserted through the anus to the descending colon and fixed caudally (approximately 2 cm from the anus). The abdominal withdrawal reflex measurements were performed 30 min after the mice were awake. After injecting 0.1 and 0.25 mL of air into the lumen of the catheter using a disposable syringe, the mice were observed for their response to rectal dilatation and scored: 0, no response; 1, rapid head movement followed by cessation of movement; 2, contraction of the abdominal muscles; 3, abdominal uplift; and 4, pelvic uplift. Each measurement was repeated 3 times with an interval of 30 s. After the test, each mouse was subjected to a visceral pain threshold test, which was performed by slowly injecting air until the mice stopped the injection when the nociceptive score was 3. The amount of air injected was recorded as the visceral pain threshold.

### 4.16. Sedation Assessment

The sedation of the mice was scored according to the Sedation Scoring Scale [44]: 4: awake, active: full movement, head up or head movement. 3: awake, inactive: eyes open, head up, no movement or head movement, normal posture. 2: slightly sedated: eyes almost closed, head slightly lowered. 1: deeply sedated: eyes closed, head completely lowered, limbs in non-routine posture. 0: asleep, completely asleep, eyes closed, body relaxed.

### 4.17. Righting Reflex

The righting reflex, which assesses sedation and depth of anesthesia in mice 1 h after the administration of the drug, demonstrates whether Dex produces anesthesia in mice.

### 4.18. Weight and Food Intake Assessment

Changes in weight of mice in each group were recorded on the evening of the day before modeling, on the day of modeling, and within 5 days after drug administration. Measurements were taken at the same time point each day.

### 4.19. Open Field Test

Tests were performed in the open field at 12 and 72 h after drug administration to AIVP mice. The testing environment was a quiet and dark box with a length of 100 cm, a width of 100 cm, and a height of 50 cm, which was divided into a central area and a peripheral area from the center point outward. After placing the mice at the center point, time was counted. The trajectory of the mice and the movement distance of each active zone were recorded for 10 min, which could reflect the level of sedation. After each mouse was tested, the stool in the dark box was cleaned and anhydrous ethanol was sprayed to remove the odor of the previous mouse.

### 4.20. Statistical Analyses

Mice were randomly assigned to different groups for the experiment. All pain tests and behavioral assessments on the experimenter’s state were performed in a blinded manner. Unless otherwise stated, data conforming to normal distribution are expressed as the mean ± standard deviation. A two-tailed Student’s *t*-test was used to analyze the statistical significance of the differences between the two groups. When comparing three or more groups, one way ANOVA with a Tukey–Kramer post hoc test was performed to analyze the comparisons, with *p* < 0.05 being considered statistically significant.

## Data Availability

Data are available on request.

## Supplementary Materials

The following supporting information can be downloaded at: https://www.mdpi.com/article/10.3390/gels11110904/s1, Table S1: Statistical reporting.

## Abbreviations

The following abbreviations are used in this manuscript:

AIVP	Acute inflammatory visceral pain
Dex	Dexmedetomidine
HA	Hyaluronic acid
MN/Dex	Dexmedetomidine dissolving microneedles
HA-MN	HA hydrogel microneedle system
NSAIDs	Nonsteroidal anti-inflammatory drugs
ERK	Extracellular signal-regulated protein kinase
TLR	Toll-like receptor
TRPV1	Transient receptor potential vanilloid 1
NF-κB	Nuclear transcription factor-κB
PVP K90	polyvinyl pyrrolidone K90
SEM	Scanning electron microscope
HUVECs	Human umbilical vein endothelial cells
RAW 264.7	Mouse monocyte-macrophage leukemia cells
Injection/Dex	Subcutaneous Dex group
Injection/Blank	Subcutaneous saline group
MN/Blank	Blank microneedles
LC-MS/MS	Liquid chromatography–tandem mass spectrometry
